# Field Performance of Bt Eggplants (*Solanum melongena* L.) in the Philippines: Cry1Ac Expression and Control of the Eggplant Fruit and Shoot Borer (*Leucinodes orbonalis* Guenée)

**DOI:** 10.1371/journal.pone.0157498

**Published:** 2016-06-20

**Authors:** Desiree M. Hautea, Lourdes D. Taylo, Anna Pauleen L. Masanga, Maria Luz J. Sison, Josefina O. Narciso, Reynaldo B. Quilloy, Randy A. Hautea, Frank A. Shotkoski, Anthony M. Shelton

**Affiliations:** 1 Institute of Plant Breeding/CSC, College of Agriculture, University of the Philippines Los Baños, College, Laguna, 4031, Philippines; 2 International Service for the Acquisition of Agri-Biotech Applications, Los Baños, Laguna, 4030, Philippines; 3 International Programs, Cornell University, Ithaca, New York, 14853, United States of America; 4 Department of Entomology, Cornell/NYSAES, Geneva, New York, 14456, United States of America; University of Tennessee, UNITED STATES

## Abstract

Plants expressing Cry proteins from the bacterium, *Bacillus thuringiensis* (Bt), have become a major tactic for controlling insect pests in maize and cotton globally. However, there are few Bt vegetable crops. Eggplant (*Solanum melongena*) is a popular vegetable grown throughout Asia that is heavily treated with insecticides to control the eggplant fruit and shoot borer, *Leucinodes orbonalis* (EFSB). Herein we provide the first publicly available data on field performance in Asia of eggplant engineered to produce the Cry1Ac protein. Replicated field trials with five Bt eggplant open-pollinated (OP) lines from transformation event EE-1 and their non-Bt comparators were conducted over three cropping seasons in the Philippines from 2010–2012. Field trials documented levels of Cry1Ac protein expressed in plants and evaluated their efficacy against the primary target pest, EFSB. Cry1Ac concentrations ranged from 0.75–24.7 ppm dry weight with the highest in the terminal leaves (or shoots) and the lowest in the roots. Cry1Ac levels significantly increased from the vegetative to the reproductive stage. Bt eggplant lines demonstrated excellent control of EFSB. Pairwise analysis of means detected highly significant differences between Bt eggplant lines and their non-Bt comparators for all field efficacy parameters tested. Bt eggplant lines demonstrated high levels of control of EFSB shoot damage (98.6–100%) and fruit damage (98.1–99.7%) and reduced EFSB larval infestation (95.8–99.3%) under the most severe pest pressure during trial 2. Moths that emerged from larvae collected from Bt plants in the field and reared in their Bt eggplant hosts did not produce viable eggs or offspring. These results demonstrate that Bt eggplant lines containing Cry1Ac event EE-1 provide outstanding control of EFSB and can dramatically reduce the need for conventional insecticides.

## Introduction

Since their introduction in 1996, maize and cotton expressing insecticidal proteins derived from the soil bacterium *Bacillus thuringiensis* (Bt) have been widely adopted and in 2014 were planted on 78.8 million ha in 28 countries predominantly by resource-poor farmers [1]. Bt crops are another form of host plant resistance, the foundation for integrated pest management (IPM) programs [2]. Several major maize and cotton pests have been successfully controlled, and insecticide use on them has been substantially reduced throughout most adopting countries [3]. Unfortunately, the development of Bt crops has been limited to major commodity crops (maize, cotton, and soybean) and not fruit and vegetables, except sweet corn. This situation is especially unfortunate since fruit and vegetables, when taken together, receive more insecticides than maize, cotton and rice combined [4].

Eggplant, *Solanum melongena* L. (also known as brinjal and aubergine) is one of the most important, inexpensive and popular vegetable crops grown and consumed in Asia. In the Philippines, eggplant production accounts for more than 30.0% of the total volume of production of the most important vegetables in the country [5]. Eggplant production provides an important source of cash income, particularly for small, resource-poor farmers. The biggest constraint to eggplant production throughout Asia is the chronic and widespread infestation by the eggplant fruit-and-shoot borer (EFSB), *Leucinodes orbonalis* Guenée [6]. The larvae damage eggplant by boring into the petiole and midrib of leaves and tender shoots resulting in wilting and desiccation of stems. Flowers are also fed upon resulting in flower drop or misshapen fruits. The most serious economic damage caused by EFSB is to the fruit by producing holes, feeding tunnels and frass (or larval excrement) that make the fruit unmarketable and unfit for human consumption. At high pest pressure, EFSB damage in the Philippines results in yield loss of up to 80.0% of the crop [7]. Surveys of eggplant farmers in the major eggplant growing provinces of the Philippines [7–11] revealed that almost all of them use chemical insecticides to control EFSB because other control measures such as manual removal of EFSB-damaged fruits and wilted shoots, use of biological control arthropods and pheromone traps [12] have proven ineffective, impractical and expensive. Eggplant farmers in the Philippines employ frequent applications (20–72 times for 5–6 months/season) of mixtures of insecticides to control EFSB, which increase production costs and pose risks to human health and the environment. Studies conducted in Sta. Maria, Pangasinan [8,13,14] showed frequent use of broad-spectrum insecticides including profenofos, triazophos, chlorpyrifos, cypermethrin, and malathion. Residues of these insecticides were detected in the soil of eggplant farms and in harvested fruits [14]. Farmers and farm workers in the study attributed various ailments such as skin irritation, redness of the eyes, muscle pains and headaches to exposure to these pesticides.

After more than 40 years, conventional breeding has not produced any commercial variety of eggplant conferring high level of resistance to the EFSB [15]. Therefore, efforts became focused on developing Bt eggplant that expresses the same Cry1Ac protein as the cotton event MON531, which has been approved by regulatory agencies in many countries [16–18]. MON531 has been bred into cotton varieties that have been on the global market for almost 20 years with no verifiable report of any adverse effect on human health or the environment. The modified gene used in MON531 encodes an amino acid sequence that is 99.4% identical to the naturally occurring microbial Cry1Ac protein [19,20].

Maharashtra Hybrid Seeds Co. Pvt. Ltd. (Mahyco) inserted the *cry1Ac* gene under the control of the constitutive 35S CaMV promoter into eggplant to control feeding damage caused by EFSB [21]. The transformation event designated as 'EE-1' was introgressed into eggplant varieties and hybrids in India, Bangladesh and the Philippines [22,23]. In 2009, although the Indian biosafety regulatory agency gave biosafety approval to Mahyco event EE-1, the Ministry of the Environment and Forests placed a moratorium on its cultivation in India [24] that remains in effect as of May 2016. In 2013, four Bt eggplant varieties containing the same EE-1 event were conditionally approved for cultivation in Bangladesh. These were grown on 20 fields in 2014 and the number increased to 108 farms in 2015 (https://bteggplant.wordpress.com/2015/08/11/speech-by-dr-md-rafiqul-islam-mondal-director-general-bari/). In the Philippines, event EE-1 was introgressed into selected EFSB-susceptible eggplant open-pollinated (OP) varieties through conventional backcrossing coupled with diagnostic EE-1 event-specific PCR and a *cry1Ac* gene strip assay [25]. Five promising advanced Bt OP lines, developed by the University of the Philippines Los Baños, were selected for Confined Field Trial testing in selected eggplant growing areas of the country.

The studies presented in this report contain the first data on Bt eggplant for control of EFSB in Asia to be submitted to a peer-reviewed journal. The studies were conducted with the following objectives: (1) to determine the expression levels of Cry1Ac protein in Bt eggplant OP lines; and (2) to evaluate the field efficacy of the EE-1 event in Bt eggplant OP lines against field populations of EFSB. Results of these studies will be used to generate crucial information for selecting the best EFSB-resistant Bt eggplant OP lines for market release in the Philippines.

## Results

### Bt Cry1Ac protein expression in different plant parts in Bt Eggplant OP lines

**Fig 1:**
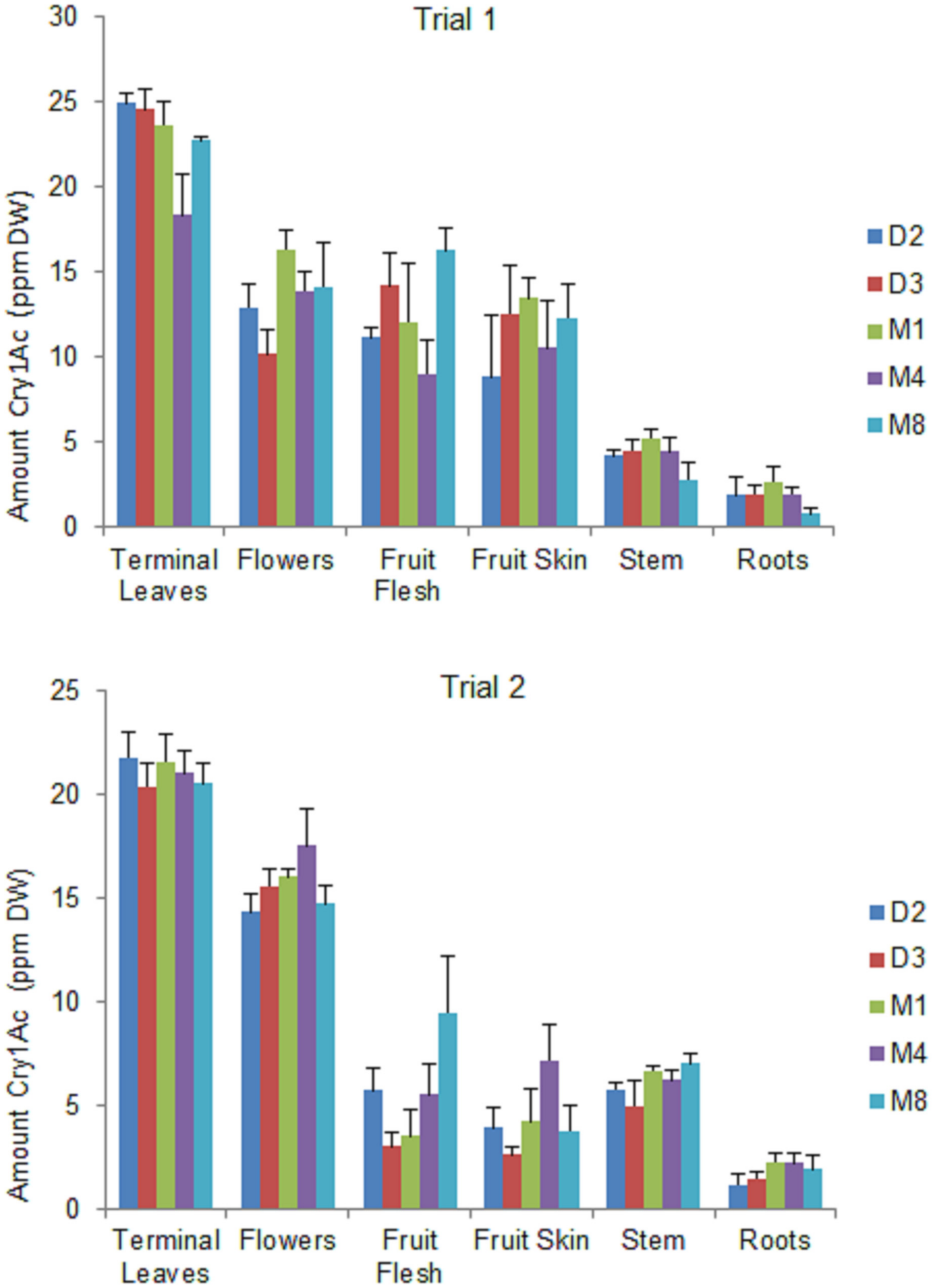
Variation in Cry1Ac protein expression in different plant parts of Bt eggplant OP lines. Mean ± SEM Cry1Ac protein concentration in terminal leaves, flowers, fruits, stem, and roots of five (5) Bt eggplant OP lines. n = 4 per plant part/Bt line with 5–10 sample plants per replicate plot. Confined field trial1 (wet/off season) and trial 2 (dry season), CY 2010–2011, Pangasinan, Philippines.

Significant differences were detected in Cry1Ac protein expression among the different plant parts in all Bt eggplant OP lines grown for two seasons (Fig 1, S1 Table). The highest levels of Cry1Ac protein were detected in the terminal leaves, with decreasing levels of expression in the flowers, fruits, stem and roots. Results of the gene strip test of the non-Bt eggplant comparators (near-isoline counterparts and check) were negative and the quantitative ELISA values were below the limit of quantitation (LOQ = 0.125) of the assay used.

#### Terminal Leaves

The Cry1Ac protein expressed in terminal leaves ranged from 18.32–24.87 ppm dry weight (DW) in trial 1, and 20.40–21.83 ppm DW in trial 2. Line M4 expressed significantly less Cry1Ac protein than line D2 in trial 1; however there were no other significant differences in expression levels among the Bt eggplant lines in either trial.

#### Flowers

There were no significant differences detected in Cry1Ac protein content among the Bt eggplant lines in both trials. Cry1Ac protein content in the flowers ranged from 10.17–16.33 ppm DW in trial 1, and from 14.34–17.57 ppm DW in trial 2.

#### Fruits (flesh and skin)

There were no significant differences in Cry1Ac protein expression in either the fruit flesh or the skin among the Bt eggplant lines tested in both trials. However, the widest range of variation was observed between the two trials. In trial 1, the fruit flesh contained higher levels of Cry1Ac protein at 9.00–16.23 ppm DW and the fruit skin ranged from 8.82–13.42 ppm DW, but much lower levels of Cry1Ac protein were detected in both the flesh and skin in trial 2 (3.02–9.47 and 2.61–7.18 ppm DW, respectively).

#### Stem

There were no significant differences in Cry1Ac protein expression in the stem among all Bt eggplant lines tested. The stem contained Cry1Ac protein concentration of 2.75–5.22 ppm DW in trial 1 and 5.00–7.02 ppm DW in trial 2.

#### Roots

The roots contained the lowest levels of Cry1Ac protein. The mean Cry1Ac protein concentration (1.8 ppm DW) was similar in both trials. There were no significant differences in Cry1Ac protein expression in the roots among the Bt eggplant lines tested. The highest level of Cry1Ac protein expressed in the roots was 2.64 ppm DW.

### Cry1Ac protein expression in terminal leaves at different growth stages in Bt Eggplant OP lines

**Fig 2:**
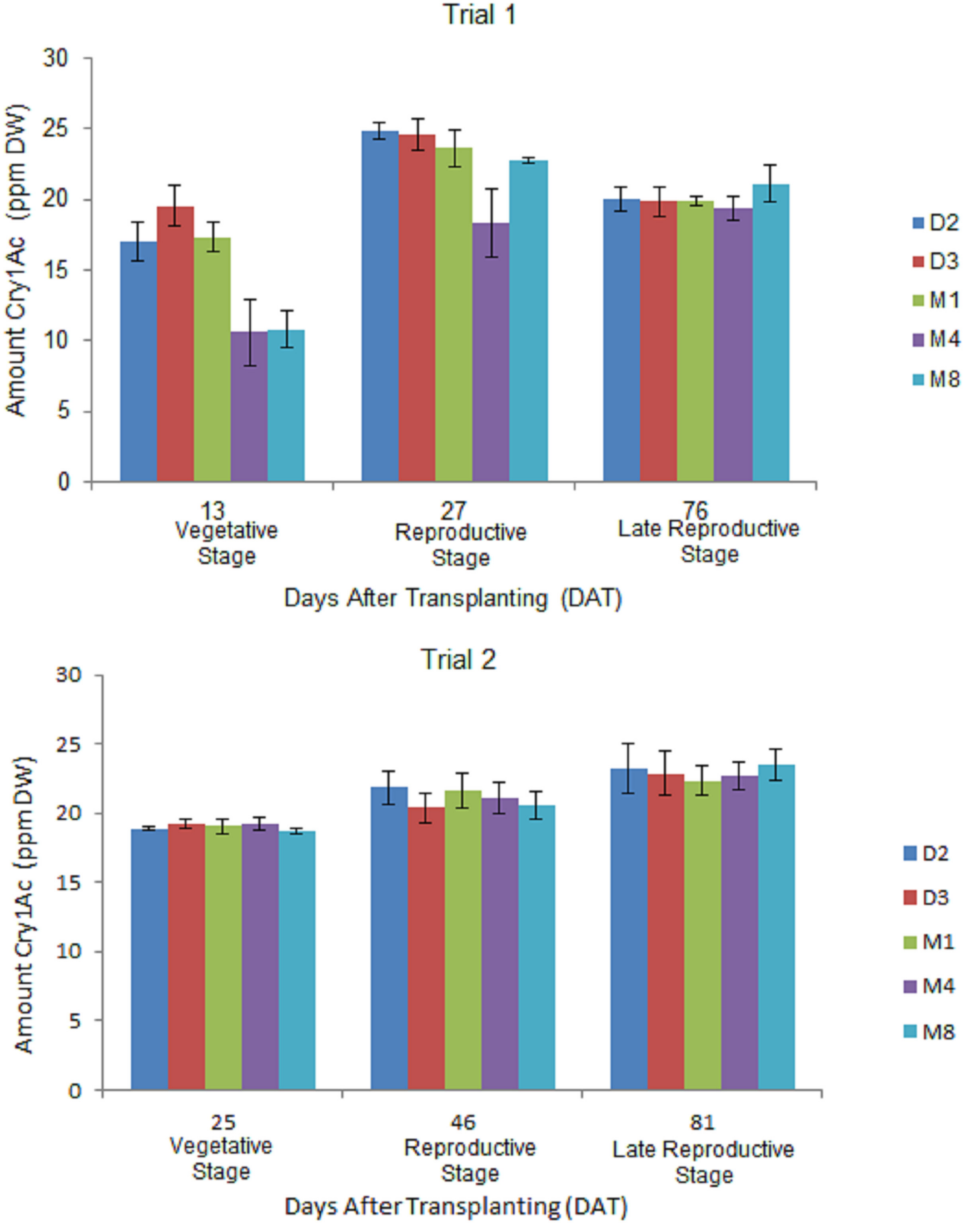
Temporal variation in Cry1Ac protein expression terminal leaves of in Bt eggplant OP lines. Mean ± SEM Cry1Ac protein concentration in the terminal leaves of 5 Bt eggplant OP lines at the vegetative, reproductive and late reproductive stages. n = 4 per growth stage/line with 5–10 plants per replicate plot. Confined field trial 1 (wet/off season) and trial 2 (dry season), CY 2010–2011, Pangasinan, Philippines.

Significant differences in Cry1Ac protein expression were detected in terminal leaves across three growth stages of eggplant development in all Bt eggplant OP lines in both trials (Fig 2, S2 Table). The observed pattern of Cry1Ac protein expression generally increased from the vegetative stage to the reproductive stage; then at the late reproductive stage the levels slightly decreased in trial 1 but increased in trial 2. Higher concentrations of Cry1Ac protein were detected in trial 2 at the vegetative stage (18.69–19.22 ppm DW) and late reproductive stage (22.32–23.54 ppm DW) compared with amounts detected at the same growth stages during trial 1. Significant differences were observed among the Bt eggplant lines in the amount of Cry1Ac protein expressed during the vegetative and reproductive stages in trial 1. Bt eggplant line M4 showed the lowest level of Cry1Ac protein expression among the lines tested. However, no significant differences were observed among the Bt eggplant lines at all growth stages in trial 2.

### Control of EFSB by Bt Eggplant OP lines

**Fig 3:**
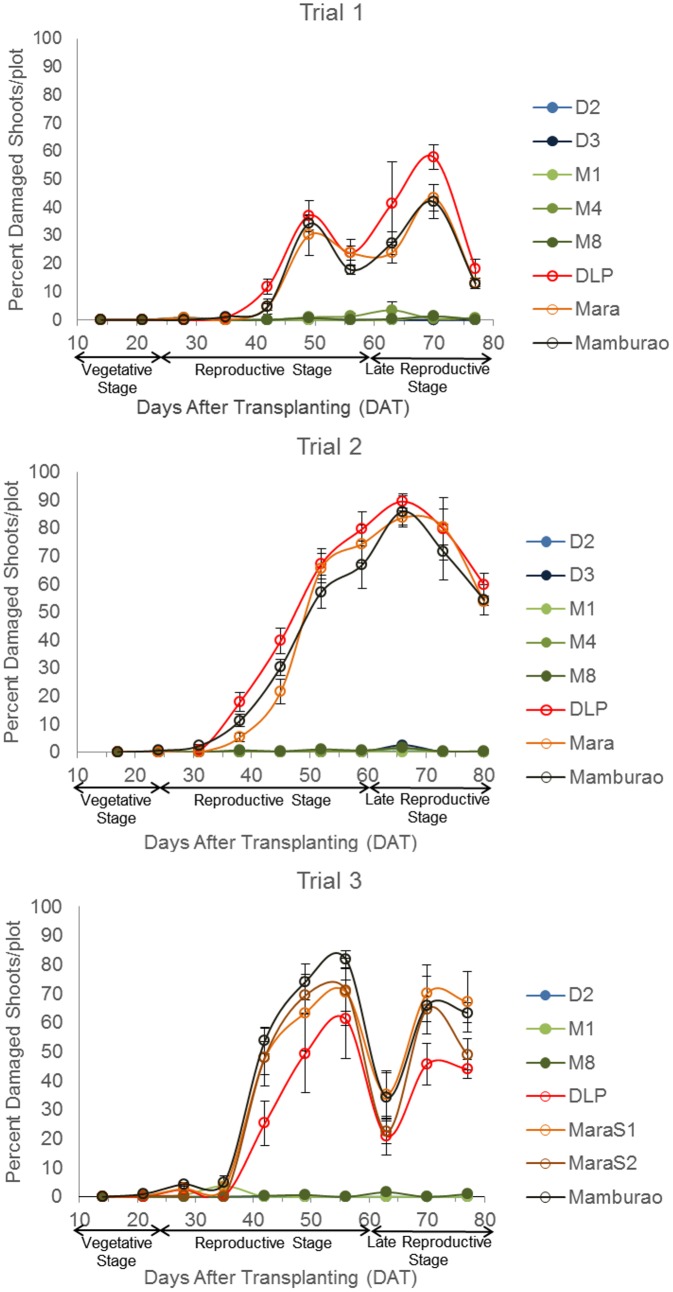
Temporal variation in % shoot damage in Bt vs. non Bt eggplants. Mean ± SEM percentage (%) EFSB-damaged shoots in Bt lines and their non-Bt eggplant comparators at different sampling periods. n = 4 per entry with 16 plants per replicate plot. Confined field trials 1 and 3 (wet/off season) and trial 2 (dry season), CY 2010–2012, Pangasinan, Philippines.

**Fig 4:**
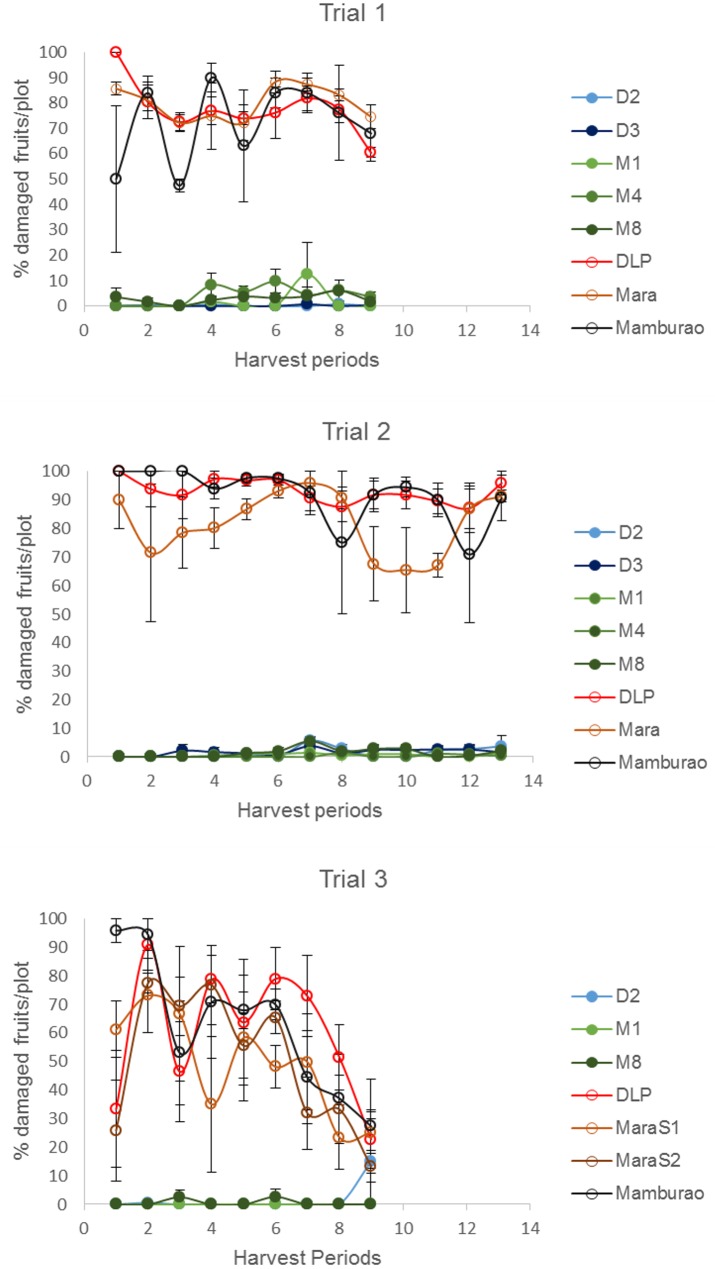
Temporal variation in % fruit damage in Bt vs. non Bt eggplants. Mean ± SEM percentage (%) EFSB-damaged fruits in Bt lines and their non-Bt eggplant comparators at different harvest periods. n = 4 per entry with 16 plants per replicate plot. Confined field trials 1 and 3 (wet/off season) and trial 2 (dry season), CY 2010–2012, Pangasinan, Philippines.

**Fig 5:**
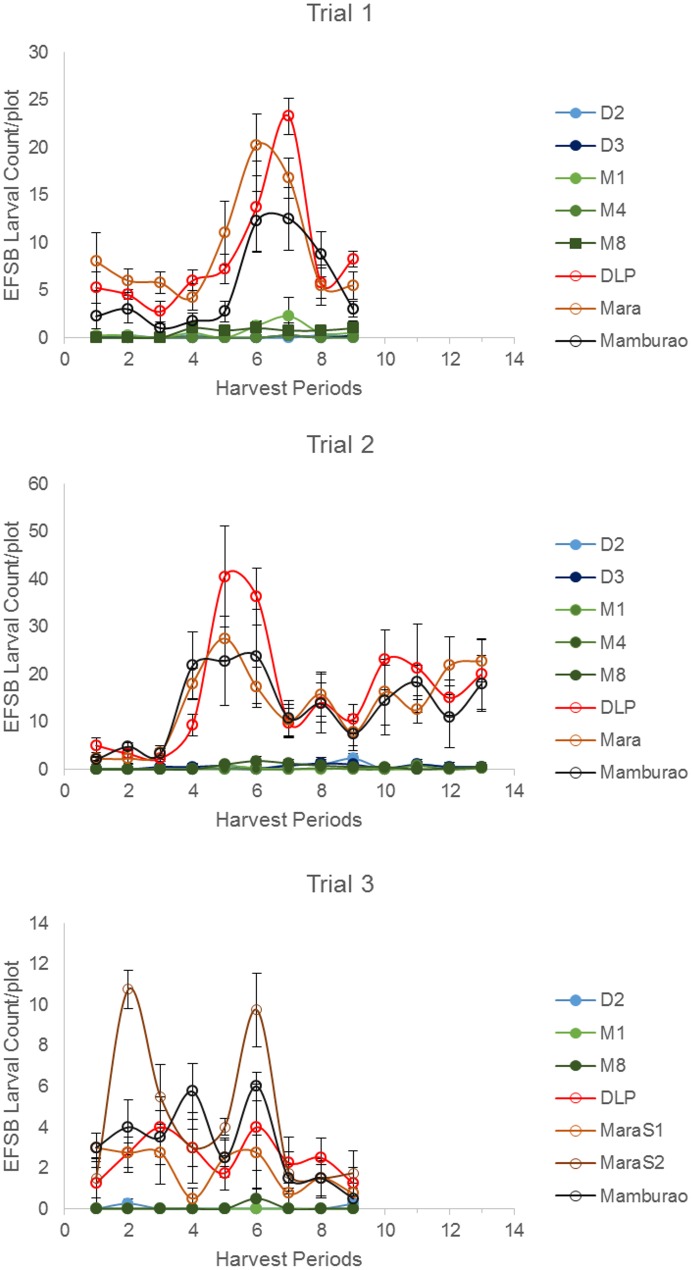
Temporal variation in number of surviving EFSB larvae in Bt vs. non Bt eggplants. Mean ± SEM number of EFSB larvae in damaged fruits of Bt lines and their non-Bt eggplant comparators at different harvest periods. n = 4 per entry with 16 plants per replicate plot. Confined field trials 1 and 3 (wet/off season) and trial 2 (dry season), CY 2010–2012, Pangasinan, Philippines.

Under natural field infestations, the efficacy against EFSB of Bt eggplant lines and the non-Bt comparators (near-isoline counterparts and check variety) were evaluated for three seasons (trials 1–3) based on the following parameters: % EFSB-damaged shoots, % EFSB-damaged fruits and number of EFSB larvae in fruits. Throughout the sampling/harvest periods, Bt eggplant lines consistently demonstrated a lower percentage of EFSB-damaged shoots (Fig 3, S3 Table), % EFSB- damaged fruits (Fig 4, S4A to S4C Table) and number of EFSB larvae in fruits (Fig 5, S4A to S4C Table) compared to the conventionally-bred non-Bt eggplant comparators.

Significant differences among entries (*P = <0*.*0001*) were detected in all parameters measured in the three trials. Results of paired mean comparison by contrast for all parameters and corresponding level of control (% efficacy) relative to non-Bt eggplant are presented in Table 1. Highly significant differences were consistently detected between Bt eggplant lines and their corresponding non-Bt eggplant comparators for all parameters in every trial. Comparisons between Bt eggplant lines and non-Bt eggplant comparators showed significantly lower shoot and fruit damage and fewer surviving EFSB larvae in fruits in the Bt eggplant lines tested. Bt eggplant demonstrated 97.7–100% and 96.0–100% control of EFSB shoot and fruit damage, respectively, except in Bt line M4 in trial 1. This line had slightly lower % efficacy for shoot damage (95.3%) and fruit damage (94.1%) but these levels were significanly much better than any of the the non-Bt comparators. Control of EFSB larval infestation in Bt eggplant lines ranged from 88.4–100%, with most lines showing > 96.0% control, except for Bt M1 and M8 in trial 1. Nevertheless, the levels of control of EFSB larval infestation in M1 and M8 were still far better compared to any of the non-Bt comparators tested.

**Table 1:** Mean comparison^1^ of % EFSB-damaged shoots, % damaged fruits, EFSB larval count and percentage efficacy between Bt eggplant (OP) lines and non-Bt (NBt) eggplant comparators.

Trial	Contrast Bt vs Non-Bt^2^	% Damaged shoots/plot^3^			% Efficacy^6^	% Damaged fruits/plot^4^			% Efficacy^6^	EFSB larval count/plot^5^			% Efficacy^6^
		Bt	NBt			Bt	NBt			Bt	NBt		
**1**	**D2 vs DLP**	0.17	19.19	**	99.1	0.19	77.76	**	99.8	0.08	8.53	**	99.0
	**D3 vs DLP**	0.00	19.19	**	100	0.12	77.76	**	99.8	0.06	8.53	**	99.4
	**M1 vs Mara**	0.14	13.99	**	99.0	1.57	79.93	**	98.1	0.61	9.22	**	93.4
	**M4 vs Mara**	0.66	13.99	**	95.3	4.21	79.93	**	94.7	0.03	9.22	**	99.7
	**M8 vs Mara**	0.32	13.99	**	97.7	2.91	79.93	**	96.4	0.58	9.22	**	93.7
	**D2 vs Mamburao**	0.17	14.14	**	98.8	0.19	71.85	**	99.7	0.08	5.25	**	98.4
	**D3 vs Mamburao**	0.00	14.14	**	100	0.12	71.85	**	99.8	0.06	5.25	**	98.9
	**M1 vs Mamburao**	0.14	14.14	**	99.0	1.57	71.85	**	97.8	0.61	5.25	**	88.4
	**M4 vs Mamburao**	0.66	14.14	**	95.3	4.21	71.85	**	94.1	0.03	5.25	**	99.4
	**M8 vs Mamburao**	0.32	14.14	**	97.7	2.91	71.85	**	96.0	0.58	5.25	**	89.0
**2**	**D2 vs DLP**	0.04	41.58	**	99.9	1.42	93.08	**	98.5	0.52	16.15	**	96.8
	**D3 vs DLP**	0.28	41.58	**	99.3	1.74	93.08	**	98.1	0.56	16.15	**	96.6
	**M1 vs Mara**	0.00	36.77	**	100	0.62	81.89	**	99.2	0.12	13.67	**	99.2
	**M4 vs Mara**	0.21	36.77	**	99.4	0.28	81.89	**	99.7	0.10	13.67	**	99.3
	**M8 vs Mara**	0.49	36.77	**	98.8	1.47	81.89	**	98.2	0.50	13.67	**	96.3
	**D2 vs Mamburao**	0.04	36.20	**	99.9	1.42	91.82	**	98.4	0.52	13.23	**	96.1
	**D3 vs Mamburao**	0.28	36.20	**	99.2	1.74	91.82	**	98.1	0.56	13.23	**	95.8
	**M1 vs Mamburao**	0.00	36.20	**	100	0.62	91.82	**	99.3	0.12	13.23	**	99.1
	**M4 vs Mamburao**	0.21	36.20	**	99.4	0.28	91.82	**	99.7	0.10	13.23	**	99.3
	**M8 vs Mamburao**	0.49	36.20	**	98.6	1.47	91.82	**	98.4	0.50	13.23	**	96.2
**3**	**D2 vs DLP**	0.00	24.56	**	100	1.73	59.94	**	97.1	0.06	2.53	**	97.6
	**M1 vs Mara S1**	0.44	36.06	**	98.8	0.00	49.00	**	100	0.00	1.92	**	100
	**M8 vs Mara S1**	0.34	36.06	**	99.1	0.57	49.00	**	98.8	0.06	1.92	**	96.9
	**M1 vs Mara S2**	0.44	32.56	**	98.6	0.00	49.89	**	100	0.00	4.39	**	100
	**M8 vs Mara S2**	0.34	32.56	**	99.0	0.57	49.89	**	98.9	0.06	4.39	**	98.6
	**D2 vs Mamburao**	0.00	38.38	**	100	1.73	62.34	**	97.2	0.06	3.14	**	98.1
	**M1 vs Mamburao**	0.44	38.38	**	98.8	0.00	62.34	**	100	0.00	3.14	**	100
	**M8 vs Mamburao**	0.34	38.38	**	99.1	0.57	62.34	**	99.1	0.06	3.14	**	98.1

^1^ Mean comparison by contrast (PROC MIXED in SAS);

** highly significant at 1% probability level; mean of 4 replicates;

^2^ Bt = eggplant containing event ‘EE-1’; NBt = non-Bt eggplant near-isolines and commercial check;

^3^ Mean of 10 weekly observation periods;

^4^ Mean of total harvest periods: Trial 1 (9 harvests); Trial 2 (13 harvests); Trial 3 (9 harvests)

^5^ Mean of total harvest periods: Trial 1 (9 harvests); Trial 2 (13 harvests); Trial 3 (9 harvests)

^6^%Efficacy = (1-Bt/non-Bt) x 100

Seasonal variation in field damage was also observed between Bt eggplant lines and their non-Bt comparators (Table 1). Trials 1 and 3 were conducted during the wet/off planting season when most of the surrounding annual crop was rice. Trial 2 was conducted during the dry season, when eggplants are more widely grown in Pangasinan. Of the three trials conducted, the highest pest pressure was recorded during trial 2 as evidenced by the highest percentages of plant damage and number of insects observed. During trial 2, the highest mean % EFSB-damaged shoots (41.58%), % EFSB-damaged fruits (93.08%), and number of surviving EFSB larvae (16.15 larvae/plot/harvest) were recorded in the non-Bt eggplant comparators. Under such severe pest pressure, the Bt eggplant lines showed <1% EFSB shoot damage, <2% fruit damage and fewer EFSB larvae (<1 larva/plot/harvest).

### Survival and Fecundity of EFSB in Bt Eggplant OP lines

EFSB larvae were collected from plants of Bt eggplant lines and non-Bt comparators and brought to the Entomology laboratory and reared continuously in their respective hosts. The results showed that very few EFSB larvae were collected in all Bt eggplant compared with the non-Bt eggplant plants sampled (Table 2). Of the total (27) EFSB larvae reared from Bt eggplant plants, less than half (11/27) emerged as adults and almost half (5/11) of the moths were weak and died before mating. Only six adults were able to mate successfully. However, no viable eggs and offspring resulted from any paired matings involving either male or female EFSB adults collected and reared in the Bt eggplants (Table 3). In contrast, a high percentage (97.3%) of EFSB larvae collected from the non-Bt plants successfully emerged as adults, mated and produced many viable eggs and young larvae.

**Table 2:** Development and survivorship of EFSB larvae collected in Bt OP lines and non-Bt eggplants^1^

Crop Type^2^	Number of field collected larvae^3^	Number of developed pupae	Number of adult emerged	No. of adults survived/mated	Physiological condition of emerged adults
**Bt OP lines**					
Total	27	17	11	6	6 normal; 5 weak
Mean^4^	0.19	0.12	0.08	0.04	
Relative %^5^	6.1	5.5	4.9	2.7	
**Non-Bt Counterparts**					
Total	415	294	215	215	all normal
Mean^4^	7.41	5.25	3.84	3.84	
Relative %^5^	93.9	94.5	95.1	97.3	
**Bt + non-Bt**					
Total	442	311	226	221	

^1^EFSB larvae collected from Bt and non-Bt hosts from larvae collected from trial 2 in Pangasinan and reared continuously on respective hosts in the IPB Entomology P2 Laboratory

^2^Bt = eggplant containing event EE-1; NBt = non-Bt eggplant counterpart genotypes

^3^ Total larval counts collected in 5 Bt OP lines and 2 non-Bt counterparts; 4 reps and 7 harvests

^4^Mean of 5 Bt OP lines and 2 non-Bt OP counterpart lines; 4 reps and 7 harvests

^5^Relative % = (Total Bt)/(Total (Bt + Non-Bt)) x 100; (Total Non-Bt)/(Total (Bt + Non-Bt)) x 100

**Table 3:** Survival and reproduction in paired matings of EFSB adults collected from Bt and non-Bt eggplants.

Mating combination^1^	No. Pairs^2^	No. eggs laid		No. neonates	
		Mean	Range	Mean	Range
**Bt ♀: NBt ♂**	4	0.00	-	0.00	-
**NBt ♀: Bt ♂**	2	0.00	-	0.00	-
**NBt ♀: NBt ♂ (control)**	2	45.00	36–54	27.50	24–31

^1^Bt = eggplant containing event EE-1; NBt = non-Bt eggplant counterpart genotypes

^2^Paired matings included all surviving adults collected from Bt hosts reared continuously on same hosts; paired mating of the control were representative samples of surviving adults collected from non-Bt hosts reared continuously on the same host

## Discussion

### Spatio-Temporal Expression of Cry1Ac Protein in Bt Eggplant Lines

Recent reviews [18, 26,27] of Bt crops engineered to express δ-endotoxin proteins cited numerous reports indicating that the expression of Cry proteins vary with plant parts, plant age, genotypes and environmental conditions. To provide the greatest benefits, Cry proteins should be expressed in sufficient quantities to provide high levels of protection to appropriate plant parts and at the stage of growth when the target insect pest pressure is most severe. In this study, significant differences were detected in the amount of Cry1Ac expressed in different plant parts: terminal leaves > flowers > fruits > stem > roots. Cry1Ac expression in the pollen was below the limit of quantitation (LOQ) of the assay used (LOQ = 0.125) (unpublished greenhouse data). It is noteworthy that higher amounts were detected in plant parts preferably attacked by the primary target pest, EFSB. The level of expression of Cry1Ac in Bt eggplant lines tested varied between 0.75±0.33 to 24.87±0.56 ppm DW. These findings are consistent with previous studies conducted in the Philippines [25] and India [21,28] showing that Bt eggplants have higher levels of Cry1Ac protein expressed in the terminal leaves, flowers and fruits than in the stem and roots. Similarly, a number of studies conducted in other countries also reported variability in Cry protein expression in plant parts in other Bt crops including cotton [29–31], corn [32–34] and rice [35].

Many researchers have also reported variation in Cry1Ac protein expression in Bt cotton during the growth and development of the plant [36–42]. In Bt cotton, Cry1Ac protein levels were generally high at early stages and then declined as the plant grew to maturity [31, 43]. In this study, seasonal variation was also detected in the level of Cry1Ac protein expression in the terminal leaves of Bt eggplant lines. However, the amount of Cry1Ac protein expressed varied only up to 1.7-fold throughout the growing season of 120 days required for profitable eggplant production. Contrary to the results in Bt cotton, in this study the level of Cry1Ac protein expression significantly increased from the vegetative stage to the reproductive stage and either slightly declined or increased at the late reproductive stage depending on the trial. It is important to note that the amount of Cry1Ac protein expressed in Bt eggplant OP lines peaked during the fruit-bearing stage and remained high with the average at 20–23 ppm DW as EFSB pest pressure became more severe.

### Factors Affecting Variability in Cry1Ac Protein Expression in Bt Eggplant Lines

Data from other crops also suggest that factors inherent to the variety and the environment affect the variability of Cry1Ac expression. These factors include among others, transgene promoter, parental background, and environmental stressors such as high temperature, heavy drought, waterlogging, and insect damage [38,44,45]. In this study, variability in Cry1Ac protein expression in the Bt eggplant lines could also be attributed to using the constitutive 35S CaMV promoter in the EE-1 gene construct, as suggested in studies with Bt cotton which used the same promoter. Parental background has also been reported to affect Cry1Ac protein variability in Bt cotton [30,31]. In this study, the parental background (‘Mara’ and ‘DLP’) of the Bt eggplant OP lines may have influenced, but only to a limited extent, the spatio-temporal variability in Cry1Ac expression. Finally, environmental factors could have contributed to the spatio-temporal expression of the Cry1Ac protein in Bt eggplant lines. Results showed that the levels of Cry1Ac detected during the entire growing season during trial 2 were different compared to results from trial 1. Trial 1 was conducted during the off-season eggplant planting, while trial 2 was conducted during the regular dry season planting. During trial 2, there were more eggplant planted, hence the level of EFSB pest pressure was higher during this season resulting in more damage as shown in the field efficacy data. Weather data obtained during the duration of the two trials indicated that the average daily temperature was similar but the amount of rainfall was much higher in trial 1 than trial 2. A previous report [46] suggested that environmental factors such as temperature and insect damage could influence expression of a Cry protein.

### Variation in Cry1Ac Protein Expression and its Effects on Field Efficacy of Bt Eggplant Lines

It has been a key concern for developers of Bt crops whether variation in Cry protein expression may cause variation in control of the target insect pest. A number of studies in Bt cotton showed that concentration of Cry1Ac correlates well with the efficacy against the target insect pests and that, as the amount of Cry1Ac declines when the crop matures, there is a concomitant decrease in % mortality of the target pest, bollworm (*Helicoverpa armigera* or *Helicoverpa zea*) [29,37,39,47–49]. In this study, the highest concentration of Cry1Ac protein was expressed in the terminal leaves (24.87± 0.56 ppm DW) and remained high as the Bt eggplant crop matured. The field efficacy of Bt eggplant lines, measured as % EFSB-damaged shoots, also remained very high (95.4–100% reduction) during the entire 10 weeks of evaluation. These results suggest that the high level of expression of Cry1Ac protein results in high field efficacy in Bt eggplant lines. The reduced EFSB-damaged shoots indicate that the effective control of EFSB starting at the vegetative stage will help reduce the field population of EFSB during the fruit-bearing stage resulting in much reduced EFSB damage. Among the plant parts, the level of Cry1Ac protein expressed in the fruits (flesh and skin) was intermediate (2.61±0.36–12.52±3.41 ppm DW). Nevertheless, the % EFSB-damaged fruits in Bt eggplants were effectively reduced (94.1–100% control) throughout the reproductive period of the plants. It should be noted that the lowest concentrations of Cry1Ac detected in the shoots (18.32±2.45 ppm DW) and fruits (2.61±0.36 ppm DW) in the Bt eggplant lines were well above the baseline susceptibility benchmark values of *L*. *orbonalis* for Cry1Ac previously reported from India. The average moult inhibitory concentration, MIC_95_, from 29 *L*. *orbonalis* populations tested for Cry1Ac was 0.059 ppm [21,28]. More recent work reported the baseline limits for MIC_50_ = 0.003 to 0.014 ppm and MIC_95_ = 0.028 to 0.145 ppm [50]. The median lethal concentrations reported were LC_50_ = 0.020 and 0.042 ppm [50] and LC_50_ = 0.0326 to 0.0369 mg/mL and LC_90_ = 0.0458 to 0.0483 mg/mL of diet [51]. MIC values have been used in corn as the best estimator of "functional mortality" and predictor of potential effectiveness of Bt corn [52].

### Field Efficacy of Bt Eggplant Lines Containing Event EE-1 Against the Primary Target Pest, ESFB

Efficacy is the capacity of the host plant to affect the survival of the insect pest. Host plant resistance can be measured as a percentage of damage to the foliage or fruiting parts, reduced crop stand, yield and vigor [53]. It can also be measured based on insect characteristics which include number of eggs laid, aggregation, food preference, growth rate, food utilization, mortality and longevity. In this study, the field efficacy of Bt eggplants against EFSB was evaluated based on the following parameters: (1) percentage of EFSB-damaged shoots; (2) percentage of EFSB-damaged fruits; (3) EFSB larval counts; and (4) survival and fecundity of field collected larvae.

The results of the three season trials indicated consistent, high field efficacy in all Bt eggplant lines tested relative to their non-Bt eggplant comparators i.e. non- Bt near-isoline counterparts and check variety. Even under the most severe pest pressure during trial 2, the Bt eggplant lines demonstrated high level of control of EFSB shoot damage (98.6–100%) and fruit damage (98.1–99.7%) and reduced EFSB larvae infestation (95.8–99.3%). Among the lines tested, Bt M4 showed the lowest % efficacy in shoot (95.3%) and fruit damage (94.1%) and M8 the lowest % efficacy for EFSB larval count (88.4%). However, these lower results were not consistently observed in every trial and their efficacy levels were always much better compared to any of the non-Bt comparators tested.

In addition to Cry1Ac expression and plant damage, we assessed the effect of Bt eggplants on EFSB survivorship and fecundity. This was done to assess the potential for evolution of resistance of EFSB to Bt eggplant. Resistance among insects occur when genetic variation in a population enables a subset of individuals to survive on doses lethal to the majority of the population when feeding on the Bt plant and subsequently produce viable offspring [54,55]. It is noteworthy that results showed few adults emerged and no eggs and viable offspring were produced in mating adults from larvae collected in Bt eggplant lines lending further evidence of very high field efficacy against EFSB. Furthermore, the diminished capacity for normal insect development and reproduction suggest the Bt eggplant lines tested in these trials express a high dose, a key component in the high dose-refuge management strategy [56].

Taken together, the results obtained from the two-year field testing in Pangasinan support the conclusion that Bt eggplant OP lines developed by the University of the Philippines Los Baños and containing event EE-1 possess a novel trait that provides outstanding control of EFSB making them superior to the conventional counterparts and the check, particularly when the pest pressure is high. Commercial production of Bt eggplant has great potential to reduce yield losses to EFSB while dramatically reducing the reliance of growers on synthetic insecticides to control this pest, reducing risks to the environment, to worker's health, and to the consumer [7,8,10,57].

Before Bt eggplant seeds are made available for commercial propagation, it is essential to develop an insect resistance management (IRM) plan to manage the risk of resistance evolution in the target pest. The use of high-dose/refuge strategy has been postulated to delay the potential evolution of insect resistance to the Bt crops by maintaining insect susceptibility [56]. This has been implemented for Bt cotton and Bt corn and the same needs to be extended to Bt eggplants. Some of the key elements in an IRM strategy include information on the expression profile of an insecticidal protein in the Bt crop, the inherent susceptibility of the insect, the number and dominance of genes involved, and the availability of susceptible plants as refuge. Results of the studies presented in this paper indicate that Bt eggplant OP lines expressed the Cry1Ac protein in relevant plant parts primarily attacked by EFSB at the appropriate growth stages throughout the productive life of the crop. More importantly, the amount of Cry1Ac detected in the Bt eggplant shoots and fruits remained sufficiently high to have significant activity against EFSB when compared to the baseline limits previously reported [21,28, 50,51]. Furthermore, the Bt eggplant OP lines exhibited very high levels of field efficacy against EFSB and severely diminished the capacity of EFSB to reproduce successfully.

Prior to the commercial production of Bt eggplant in the Philippines, a structured refuge management strategy will be required. In addition to a structured refuge, the presence of many conventional non-Bt eggplant varieties and alternate wild *Solanum* hosts commonly present in uncultivated peripheral lands (i.e., unstructured refuges) will serve as a source of susceptible EFSB alleles in the population to slow the evolution of resistance in EFSB. Collectively, the results of this study suggest the possibility of a high-dose/refuge strategy for Bt eggplants. A stringent implementation of high-dose/refuge IRM plan within the context of integrated pest management (IPM) could help delay the potential development of resistance of EFSB to the Bt protein in UPLB Bt eggplant lines.

## Materials and Methods

Confined field trials were conducted in the Philippines to evaluate product performance and assess potential environmental risks of UPLB Bt eggplants compared to their non-Bt comparators i.e. non-Bt near-isoline counterparts and the commercial check or reference variety. The field testing site located in the province of Pangasinan, the Philippines, best represented the agro-climatic conditions and production practices in the largest eggplant growing region (Region I or the Ilocos Region) in the country. Pangasinan has Type 1 climate characterized by two pronounced growing seasons: dry, from November to April; wet, during the rest of the year. Eggplant cultivation in Pangasinan is higher during the dry season (DS). Farmers in Pangasinan plant eggplant after rice starting in the months of September to October (planting season) and harvest during the months of December to April. Some farmers also plant during the off-season, which starts at the end of the dry season and harvest during the early wet-season. The province of Pangasinan alone has the widest production area (18.4%) and contributes the largest volume (31.9%) of eggplant produced in the country (2005–2014) [5]. The Pangasinan field trial site represented the conditions in small-holder farmer’s fields that experience very high natural incidence of EFSB pressure compared with other trial sites.

Three replicated confined field experiments were conducted in Bgy. Paitan, Sta. Maria, for three seasons from March 2010- October 2012. These trials were conducted under natural field infestation of EFSB and without application of lepidopteran-specific insecticide sprays. The studies were conducted in a comparative manner. Bt eggplant lines were evaluated in comparison with the conventional non-Bt comparators consisting of the corresponding non-Bt counterparts with similar genetic backgrounds (recurrent parents/near-isolines) and a National Seed Industry Council (NSIC)-approved commercial open-pollinated variety (OPV) as check or reference genotype. OPVs are standard varieties, which have stable characteristics and produce seeds that will grow into plants more or less identical to their parent plants.

### Plant Materials, Experimental Design and Regulatory Conditions

The experimental materials used in the series of three confined field experiments are listed in Table 4.

**Table 4:** Bt eggplant open-pollinated (OP) lines test entries, non-Bt counterparts and check variety used in confined field trials.

Trial No.	Crop Generation^2^	Duration^3^	Bt OP lines^4^	Non-Bt counterpart OP lines^5^	Non-Bt commercial check variety ^6^
**1**	BC_3_F_4_	CY 2010 (Mar- Jul 2010)	D2,D3	DLP	Mamburao
			M1,M4,M8	Mara	
**2**	BC_3_F_5_	CY 2010–11 (Sept 2010-Mar 2011)	D2,D3	DLP	Mamburao
			M1,M4,M8	Mara	
**3**	BC_3_F_6_	CY 2012 (Mar-Oct 2012)	D2	DLP	Mamburao
			M1,M8	Mara S1,Mara S2	

^1^ unsprayed = no lepidopteran-specific insecticide applied

^2^ BC_n_ = number of backcrossing; F_n_ = filial generation

^3^ From sowing to end of fallow period

^4^ D2, D3 = promising advanced Bt OP lines developed through conventional backcross breeding between improved line selection from Dumaguete Long Purple (DLP) and Mahyco eggplant event EE-1; M1, M4, M8 = promising advanced Bt OP lines developed through conventional backcross breeding between improved line selection from cultivar Mara and Mahyco eggplant event EE-1

^5^ DLP = open-pollinated improved line selection from Dumaguete Long Purple public variety; Mara, Mara S1, Mara S2 = open-pollinated improved line selections from the Mara cultivar developed by UPLB-IPB Vegetable Breeding Division

^6^National Seed Industry Council (NSIC)-registered commercial open-pollinated eggplant variety

#### Plant materials

The Bt eggplant OP lines (D2, D3, M1, M4, M8) used as test entries in the field trials are advanced breeding lines (BC_3_F_4_ to BC_3_F_6_) derived from initial crosses of Mara selection x Mahyco elite line, ‘EE-1’ and DLP selection x Mahyco elite line, ‘EE-1’. The non-Bt comparators were: (1) DLP as the non-Bt counterpart genotype of Bt D2 and D3 OP lines; (2) Mara, Mara 1 or Mara S2 as the counterpart genotypes for M1, M4 and M8; and (3) Mamburao, a non-Bt eggplant OP variety approved by the NSIC [58] as the check or reference genotype.

#### Experimental Design and Field Layout

Each field experiment was planted in randomized complete block design (RCBD) with four replications, 4–6 rows/plot and 10 plants per row. The perimeters of each field experiment were surrounded by five rows (1 m between rows) of conventional non-Bt eggplant OP as pollen-trap plants. The experimental set up was conducted in a fenced facility with restricted access. A 200-meter radial distance isolated the field trial site from the nearest eggplants in the area.

#### Permissions

All field trials were conducted in accordance with the Department of Agriculture Administrative Order No. 8 Series of 2002 for field testing (www.biotech.da.gov.ph). The Bureau of Plant Industry (BPI) issued the corresponding Biosafety Permit for Field Testing in Bgy. Paitan, Sta. Maria, Pangasinan. The biosafety permit conditions were complied with throughout the conduct of every field experiment and associated greenhouse and laboratory activities. All sample collection and transport of materials were done under the supervision of the duly designated biosafety trial inspectors following the prescribed biosafety procedure for sample collection, handling and transport.

### Crop Establishment, Management, Harvesting and Termination

#### Seedling establishment

Seeds of UPLB Bt and non-Bt eggplant entries (treatments) were sown in pots with sterilized soil 30–34 days before transplanting. The germinated seeds were pricked (transferred individually in seedling trays), 7–8 days after sowing (DAS) and maintained inside the BL2 greenhouse at UP Los Baños. At 28–30 DAS, representative seedlings for the seed lot of each entry were tested for presence or absence of Cry1Ac using immunoassay or gene strip test kit, DesiGen Xpresstrip (DesiGen, Maharashtra, India), as described in Ripalda *et al*. [25]. Excess transgenic seedlings were disposed of properly in a disposal site inside the BL2 greenhouse. Seedlings of Bt and non-Bt eggplant test entries, check varieties and pollen traps were transported from UP Los Baños to the confined field testing site in Bgy. Paitan, Sta. Maria, Pangasinan for transplanting.

#### Cultural management

The confined field trials were managed based on the national cooperative trial guidelines for eggplant [59] and prevalent agronomic practices for eggplant growing in the region, including site preparation, tillage, and nutrient applications. Manual watering of plants was done during the first month after transplanting and shifted to overhead and/or furrow irrigation as plants grew and required greater amounts of water. At times of continuous heavy rain during the trial period, trenches or canals were dug to keep the soil near the roots from being waterlogged and to reduce the incidence of bacterial wilt infection. Staking of plants was done to provide additional support as the number and size of fruits increase and during periods of strong winds and rain. Branches were kept off the ground to prevent the fruit from becoming deformed.

#### Pest management

No lepidopteran-specific insecticide sprays were applied during the entire duration of the trials. Management of other arthropod pests and diseases was done by application of recommended IPM practices, primarily sanitation and withholding of pesticide use as long as possible to enable the proliferation of natural enemies. Whenever populations of leafhoppers and mites rose to very high level, they were controlled with the application of insecticides with reduced risk and without activity against EFSB (a.i. thiamethoxam) and sulphur, respectively.

#### Termination, disposal and fallow period

After the final harvest, each field experiment was terminated. All above- and below- ground plant parts were removed from the field and disposed of properly following the prescribed procedure indicated in the Biosafety permit. The field was plowed, irrigated and observed for volunteer plants 7, 14 and 30 and 60 days after termination. The field was kept fallow for at least 60 days after termination.

### Data Collection and Analysis

#### Determination of Cry1Ac Protein Expression

Cry1Ac protein expression study was conducted in trial 1 and trial 2, which represented the two eggplant growing seasons in Pangasinan, i.e. wet/off-season and regular dry season planting, respectively. Because trial 3 was also planted during the same season as trial 1 (wet/off-season) and for cost consideration, Cry1Ac protein analysis was not performed from samples obtained in this trial.

Sample collection used was based on the protocol previously described in Ripalda *et al*. [25]. Different plant parts from at least five plants from among the 16 plants in the two inner rows/replicate plot were collected. Terminal leaves were collected at the vegetative (up to 25 days after transplanting, DAT), reproductive (25–60 DAT) and late reproductive (60–80 DAT) stages of the crop. Flowers and immature fruits were collected during reproductive and late reproductive stages. Fruit samples were collected during the harvest period. Stem and roots were collected at termination (around 150 DAT). All samples collected were kept in an icebox and transported to the laboratory. Flesh and skin of immature fruits, but avoiding seeds, were separated in thin slices. Stems and roots were washed prior to storage. The woody portion of the stems was used for analysis. All samples were kept in a -80°C biofreezer until further processing and then freeze-dried at -60°C for 1–5 days until crisp. Dried samples from three plants per plot per plant part were bulked and placed in a 2.0 mL microfuge tube. Bulked samples were homogenized using two 6-mm steel beads and the ground samples were put in sealed containers and stored at 4°C until use.

Quantification of Cry1Ac was done through an enzyme-linked immunosorbent assay (ELISA). Commercially available quantitative ELISA kits (DesiGen Cry1Ac QuanT) specific for the Cry1Ac protein were procured from Mahyco (Maharashtra, India). Five milligrams of the powdered samples were weighed and analyzed. Chilled extraction buffer prepared as specified in the kit was added to the weighed samples. A dilute (up to 1:8) trypsinized protein extract was loaded to the pre-coated plates. Positive and negative controls and standards were prepared and loaded according to the instructions in the kit. Antibodies, wash buffer and substrate for detection (pNPP) used were also from the kit. Absorbance readings of the samples were made at 405 nm. According to the manufacturer’s instruction, the assay is considered valid when the mean absorbance reading of the blank is ≤0.246, mean absorbance reading of the standards with the highest concentration of Cry1Ac is ≥1.305, % residual of back calculated concentration of standards are 20 ng/ml– 125 ng/ml standards: ≤15%; 0.625 ng/ml standard ≤25% and R^2^ of the standard curve is ≥0.98.

Data on Cry1Ac concentrations from different plant parts and different developmental stages of the Bt eggplant lines were analyzed by one-way analysis of variance using PROC MIXED in SAS v.9.1.3 [60]. Means were separated using Tukey’s HSD at α = 0.05. Data available from the Dryad Digital repository: http://dx.doi.org/10.5061/dryad.ks131 [61]

#### Evaluation of Field Damage by EFSB

Fruits were harvested from the 16 plants located in the inner two rows (12 m^2^) of each plot. During each harvest period, the harvested fruits per plot were carefully cut open and examined for the presence of EFSB larvae or signs of EFSB damage and tunneling, sorted as with or without EFSB-damage, counted and weighed separately.

Data gathered:

- Percentage (%) damaged shoots per plot–calculated from the number of damaged shoots due to EFSB recorded from five shoots per plant from 16 inner row plants per plot at weekly intervals starting at two weeks after transplanting (WAT) for 10 observation periods.
- Percentage (%) damaged fruits per plot—calculated from the total number of EFSB-damaged fruits over the total number of fruits harvested from 16 inner row plants per plot. Harvesting was done every 3–4 days. Data were collected from 10–17 harvest periods prior to the termination of the experiment.
- EFSB larval counts (no. larvae/plot)–All harvested fruits from the 16 inner row plants per were cut open to check for the presence of EFSB larvae. The number of surviving EFSB larvae found inside the fruits per replicate plot were recorded every harvest period.
- % Efficacy (or Level of control)–calculated based on the formula (1- Bt/nonBt)*100% for % EFSB-damaged shoots, damaged fruits and EFSB larval counts
- Survivorship and fecundity–All surviving larvae collected per plot per harvest period were transferred to individual plastic cups and labeled. Each cup was provided with a slice of eggplant fruit from which the larva was collected. The cups were then brought to the UPLB-IPB Entomology P2 Laboratory and reared continuously in their respective hosts (Bt or non-Bt) until the adult stage. The number of individuals that successfully reached pupal and adult stages was recorded. Pairs of surviving adults from Bt and from conventional non-Bt lines were mated, placed in oviposition chambers and observed for egg deposition and hatching of offspring.

Data transformation was used to improve the normality of variables due to markedly skewed data or heterogeneous variances of Bt and conventional non-Bt entries. Data collected were transformed to sqrt (Y+0.5), arcsin (sqrt(Y/100)) or log_10_(Y+1) as appropriate. Transformed data on percentages damaged shoots and fruits, larval counts and feeding tunnel lengths were subjected to one-way analysis of variance and analyzed using PROC MIXED in SAS v.9.1.3 [60] and means were separated using Tukey’s HSD at α = 0.05. Pairwise mean comparisons by contrast between each Bt and its respective non-Bt counterpart and check variety were done for all parameters gathered using PROC MIXED. Data available from the Dryad Digital repository: http://dx.doi.org/10.5061/dryad.ks131 [61]

## Supporting Information

S1 Table Mean ± SEM concentration of Cry1Ac in different plant parts of Bt eggplant OP lines. Trials 1 to 2. CY 2010–11, Sta Maria, Pangasinan, Philippines.
(DOCX)

S2 Table Mean ± SEM concentration of Cry1Ac in the terminal leaves of Bt eggplant OP lines at three different growth stages. Trials 1 to 2. CY 2010–11, Sta Maria, Pangasinan, Philippines.
(DOCX)

S3 Table Mean ± SEM of percentage EFSB shoot damage of Bt OP lines and non-Bt eggplants comparators. Trials 1 to 3. CY 2010–12, Sta. Maria, Pangasinan, Philippines.
(DOCX)

S4 Table Mean ± SEM of percentage EFSB fruit damage of Bt OP lines and non-Bt eggplants comparators. A: Trial 1; B: Trial 2; C: Trial 3. CY 2010–12, Sta. Maria, Pangasinan, Philippines.
(DOCX)

S5 Table Mean ± SEM EFSB larval counts in fruits of Bt OP lines and non-Bt eggplants comparators. A: Trial 1; B: Trial 2; C: Trial 3. CY 2010–12, Sta. Maria, Pangasinan, Philippines.
(DOCX)
