# Supplementary material for: Field Performance of Bt Eggplants (Solanum melongena L.) in the Philippines: Cry1Ac Expression and Control of the Eggplant Fruit and Shoot Borer (Leucinodes orbonalis Guenée)
Source: PLoS One. 2016 Jun 20;11(6):e0157498. doi: 10.1371/journal.pone.0157498 (PMC4913932; doi:10.1371/journal.pone.0157498)
Supplement: S1 Table — Trials 1 to 2. CY 2010–11, Sta Maria, Pangasinan, Philippines. (DOCX) [file pone.0157498.s001.docx]

**S1 Table. Mean concentration of Cry1Ac expressed in different plant parts of Bt eggplant OP lines.** Trials 1 and 2 CY 2010-2011Sta. Maria, Pangasinan, Philippines

| **Trial** | **Bt OP lines** | **Mean concentration of Cry1Ac (ppm DW)^1^** | | | | | |
| --- | --- | --- | --- | --- | --- | --- | --- |
|  |  | **Terminal Leaves^2^** | **Flowers** | **Marketable Fruits** | | **Stem^3^** | **Roots^3^** |
|  |  |  |  | **Flesh** | **Skin** |  |  |
| **1** | **D2** | 24.87±0.56 a | 12.88±1.43 | 11.14±0.52 | 8.82±3.67 | 4.15±0.38 | 1.88±1.08 |
|  | **D3** | 24.54±1.14 ab | 10.17±1.43 | 14.20±1.91 | 12.55±2.88 | 4.48±0.64 | 1.93±0.50 |
|  | **M1** | 23.61±1.33 ab | 16.33±1.05 | 12.02±3.41 | 13.42±1.26 | 5.22±0.46 | 2.64±0.89 |
|  | **M4** | 18.32±2.45 b | 13.85±1.15 | 9.00±1.93 | 10.54±2.76 | 4.40±0.86 | 1.91±0.37 |
|  | **M8** | 22.72±0.19 ab | 14.12±2.57 | 16.23±1.27 | 12.30±1.96 | 2.75±1.04 | 0.75±0.33 |
|  | **Mean^4^** | 22.81±1.18a | 13.47±1.00b | 12.52±1.25b | 11.52±0.82b | 4.20±0.41c | 1.82±0.31c |
|  | **Range** | 18.32-24.87 | 10.17-16.33 | 9.00-11.14 | 8.82-13.42 | 2.75-5.22 | 0.75-2.64 |
| **2** | **D2** | 21.83±1.17 | 14.34±0.90 | 5.73±1.11 | 3.93±1.01 | 5.76±0.30 | 1.17±0.51 |
|  | **D3** | 20.40±1.09 | 15.61±0.79 | 3.02±0.71 | 2.61±0.36 | 5.00±1.17 | 1.45±0.33 |
|  | **M1** | 21.62±1.32 | 16.04±0.38 | 3.53±1.25 | 4.23±1.57 | 6.69±0.24 | 2.28±0.46 |
|  | **M4** | 21.06±1.11 | 17.57±1.76 | 5.51±1.47 | 7.18±1.68 | 6.20±0.54 | 2.23±0.50 |
|  | **M8** | 20.57±0.99 | 14.72±0.90 | 9.47±3.21 | 3.77±1.45 | 7.02±0.45 | 1.90±0.68 |
|  | **Mean^4^** | 21.10±0.28a | 15.66±0.57b | 5.46±1.14c | 4.35±0.76c | 6.13±0.36cd | 1.80±0.22d |
|  | **Range** | 20.40-21.83 | 14.34-17.57 | 3.02-9.47 | 2.61-7.18 | 5.00-7.02 | 1.17-2.28 |

^1^ Means of each line; for each plant part within a trial, means for lines with the same letter group are not significantly different at α=0.05

^2^ Cry1Ac concentration in terminal leaves at reproductive stage

^3^ OPV Trial 2 data for stem and roots were obtained from ratooned crop (one month older than main crop)

^4^Means of all lines for each plant part; within a trial means of the same letter group are not significantly different at α=0.05

LOQ = limit of quantification (LOQ=0.125); Absorbance reading of the blank was below 0.245. (R^2^ = 0.98 or better)
