# Supplementary material for: Field Performance of Bt Eggplants (Solanum melongena L.) in the Philippines: Cry1Ac Expression and Control of the Eggplant Fruit and Shoot Borer (Leucinodes orbonalis Guenée)
Source: PLoS One. 2016 Jun 20;11(6):e0157498. doi: 10.1371/journal.pone.0157498 (PMC4913932; doi:10.1371/journal.pone.0157498)
Supplement: S2 Table — Trials 1 to 2. CY 2010–11, Sta Maria, Pangasinan, Philippines. (DOCX) [file pone.0157498.s002.docx]

**S2 Table.** **Mean concentration of Cry1Ac in the terminal leaves of Bt eggplant OP lines at three different growth stages.** Trials 1 to 2. CY 2010-11, Sta Maria, Pangasinan, Philippines.

| **Trial** | **Bt OP lines** | **Mean concentration of Cry1Ac (ppm DW)^1^** | | |
| --- | --- | --- | --- | --- |
|  |  | **Vegetative Stage** | **Reproductive Stage** | **Late**  **Reproductive Stage** |
| **1** | **D2** | 16.99±1.34 ab | 24.87±0.56 a | 19.97±0.86 |
|  | **D3** | 19.54±1.44 a | 24.54±1.14 ab | 19.82±1.07 |
|  | **M1** | 17.33±1.05 a | 23.61±1.33 ab | 19.93±0.31 |
|  | **M4** | 10.58±2.34 c | 18.32±2.45 b | 19.37±0.90 |
|  | **M8** | 10.83±1.28 bc | 22.72±0.19 ab | 21.10±1.34 |
|  | **Mean^2^** | 15.05±1.83 c | 22.81±1.18 a | 20.04±0.29 b |
|  | **Ranges** | 10.58-19.54 | 18.32-24.87 | 19.37-21.10 |
| **2** | **D2** | 18.87±0.14 | 21.83±1.17 | 23.27±1.79 |
|  | **D3** | 19.18±0.34 | 20.40±1.09 | 22.86±1.59 |
|  | **M1** | 19.06±0.51 | 21.62±1.32 | 22.32±1.05 |
|  | **M4** | 19.22±0.43 | 21.06±1.11 | 22.64±1.02 |
|  | **M8** | 18.69±0.24 | 20.57±0.99 | 23.54±1.12 |
|  | **Mean^2^** | 19.00±0.10 c | 21.10±0.28 b | 22.93±0.22 a |
|  | **Ranges** | 18.69-19.22 | 20.40-21.83 | 22.32-23.54 |

^1^ Means of each entry; means of the same letter groups are not significantly different at α=0.05

^2^ Means of all entries for each stage; means of the same letter groups are not significantly different at α=0
