# Supplementary material for: Field Performance of Bt Eggplants (Solanum melongena L.) in the Philippines: Cry1Ac Expression and Control of the Eggplant Fruit and Shoot Borer (Leucinodes orbonalis Guenée)
Source: PLoS One. 2016 Jun 20;11(6):e0157498. doi: 10.1371/journal.pone.0157498 (PMC4913932; doi:10.1371/journal.pone.0157498)
Supplement: S3 Table — Trials 1 to 3. CY 2010–12, Sta. Maria, Pangasinan, Philippines. (DOCX) [file pone.0157498.s003.docx]

**S3 Table**. **Mean ± SEM of percentage EFSB shoot damage of Bt OP lines and non-Bt eggplants comparators.** Percentage EFSB shoot damage^1^ at different sampling periods (days after transplanting, DAT) in Bt eggplant OP lines containing event ‘EE-1’ and conventional non-Bt eggplant comparators. Trials 1 to 3. Bgy. Paitan, Sta. Maria, Pangasinan. Philippines

| **Trial 1** | | | | | | | | | | |
| --- | --- | --- | --- | --- | --- | --- | --- | --- | --- | --- |
| Entry | 14DAT | 21DAT | 28DAT | 35DAT | 42DAT | 49DAT | 56DAT | 63DAT | 70DAT | 77DAT |
| D2 | 0±0 | 0±0 | 0.83±0.83 | 0±0 | 0±0 | 0.667±0.667 | 0±0 | 0±0 | 0±0 | 0.21±0.21 |
| D3 | 0±0 | 0±0 | 0±0 | 0±0 | 0±0 | 0±0 | 0±0 | 0±0 | 0±0 | 0±0 |
| M1 | 0±0 | 0±0 | 0±0 | 0±0 | 0±0 | 0±0 | 0±0 | 0±0 | 1.25±1.25 | 0.28±0.16 |
| M4 | 0±0 | 0±0 | 0±0 | 0±0 | 0±0 | 0.96±0.60 | 1.25±1.25 | 3.44±3.04 | 0.31±0.31 | 0.81±0.52 |
| M8 | 0±0 | 0±0 | 0.69±0.69 | 0±0 | 0±0 | 0.63±0.63 | 0±0 | 0.31±0.31 | 1.25±0.77 | 0.15±0.14 |
| DLP | 0±0 | 0±0 | 0±0 | 0.85±0.85 | 11.69±2.64 | 37.12±5.21 | 23.75±2.60 | 41.41±14.94 | 57.90±4.38 | 18.25±3.33 |
| Mara | 0±0 | 0±0 | 0.74±0.74 | 0±0 | 4.71±2.54 | 30.14±7.27 | 23.77±4.70 | 23.98±3.61 | 43.48±4.79 | 13.05±1.82 |
| Mamburao | 0±0 | 0±0 | 0±0 | 1.15±1.49 | 4.66±2.31 | 34.23±5.79 | 17.81±3.29 | 27.39±8.00 | 42.02±11.95 | 12.92±2.58 |
| **Trial 2** | | | | | | | | | | |
| Entry | 17DAT | 23DAT | 30DAT | 37DAT | 44DAT | 51DAT | 58DAT | 65DAT | 73DAT | 78DAT |
| D2 | 0±0 | 0±0 | 0±0 | 0±0 | 0±0 | 0±0 | 0±0 | 0.31±0.31 | 0±0 | 0±0 |
| D3 | 0±0 | 0±0 | 0±0 | 0±0 | 0±0 | 0±0 | 0±0 | 2.50±1.69 | 0±0 | 0±0 |
| M1 | 0±0 | 0±0 | 0±0 | 0±0 | 0±0 | 0±0 | 0±0 | 0±0 | 0±0 | 0±0 |
| M4 | 0±0 | 0±0 | 0±0 | 0±0 | 0±0 | 0.31±0.31 | 0.31±0.31 | 1.25±0.72 | 0±0 | 0±0 |
| M8 | 0±0 | 0±0 | 0±0 | 0.63±0.63 | 0.31±0.31 | 0.98±0.64 | 0.63±0.63 | 1.56±0.94 | 0.31±0.31 | 0.31±0.31 |
| DLP | 0±0 | 0±0 | 0.63±1.25 | 17.89±3.48 | 39.69±4.40 | 67.19±5.31 | 79.69±6.00 | 89.54±2.55 | 79.63±11.19 | 59.81±3.98 |
| Mara | 0±0 | 0±0 | 0±0 | 5.31±1.48 | 21.56±4.28 | 65.63±5.27 | 74.378±6.70 | 83.75±2.55 | 80.31±6.42 | 53.75±1.35 |
| Mamburao | 0±0 | 0.51±0.51 | 2.20±0.61 | 11.25±2.22 | 30.31±2.81 | 57.19±5.87 | 66.88±8.56 | 85.93±5.60 | 71.56±10.00 | 54.38±5.41 |
| **Trial 3** | | | | | | | | | | |
| Entry | 14DAT | 21DAT | 28DT | 35DAT | 42DAT | 49DAT | 56DAT | 63DAT | 70DAT | 77DAT |
| D2 | 0±0 | 0±0 | 0±0 | 0±0 | 0±0 | 0±0 | 0±0 | 0±0 | 0±0 | 0±0 |
| M1 | 0±0 | 0±0 | 0.63±0.30 | 3.75±2.73 | 0±0 | 0±0 | 0±0 | 0±0 | 0±0 | 0±0 |
| M8 | 0±0 | 0±0 | 0±0 | 0±0 | 0.313±0.255 | 0.63±0.32 | 0±0 | 1.56±1.37 | 0±0 | 0.94±1.20 |
| DLP | 0±0 | 0±0 | 2.50±0.93 | 0.31±0.26 | 25.313±6.283 | 49.38±12.08 | 57.50±12.27 | 20.94±7.71 | 45.63±10.22 | 44.06±6.40 |
| Mara S1 | 0±0 | 0.31±0.26 | 2.81±1.34 | 3.13±0.30 | 47.813±4.590 | 63.44±11.69 | 70.63±11.47 | 35.31±9.31 | 70.00±3.86 | 67.19±20.98 |
| Mara S2 | 0±0 | 0.31±0.26 | 0.31±0.26 | 0.00±0.00 | 48.125±8.223 | 69.38±5.58 | 71.25±7.27 | 22.50±4.82 | 64.69±6.10 | 49.06±10.53 |
| Mamburao | 0±0 | 0.94±0.77 | 4.38±0.51 | 5.00±1.82 | 53.750±3.750 | 74.06±5.60 | 81.88±2.91 | 34.38±9.60 | 65.94±14.08 | 63.44±6.88 |

^1^Mean of four replicates, 10 observation periods
