# Supplementary material for: Field Performance of Bt Eggplants (Solanum melongena L.) in the Philippines: Cry1Ac Expression and Control of the Eggplant Fruit and Shoot Borer (Leucinodes orbonalis Guenée)
Source: PLoS One. 2016 Jun 20;11(6):e0157498. doi: 10.1371/journal.pone.0157498 (PMC4913932; doi:10.1371/journal.pone.0157498)
Supplement: S4 Table — A: Trial 1; B: Trial 2; C: Trial 3. CY 2010–12, Sta. Maria, Pangasinan, Philippines. (DOCX) [file pone.0157498.s004.docx]

**S4A Table.** **Mean ± SEM of percentage EFSB fruit damage of Bt OP lines and non-Bt eggplants comparators.** Percentage EFSB fruit damage^1^ every harvest period (days after transplanting, DAT) in Bt eggplant OP lines containing event ‘EE-1’ and conventional non-Bt eggplant comparators .Trial 1. Bgy. Paitan, Sta. Maria, Pangasinan, Philippines

| **Entry** | **Percentage (%) EFSB fruit damage per plot** | | | | | | | | |
| --- | --- | --- | --- | --- | --- | --- | --- | --- | --- |
|  | **1st Harvest** | **2nd Harvest** | **3rd Harvest** | **4th Harvest** | **5th Harvest** | **6th Harvest** | **7th Harvest** | **8th Harvest** | **9th Harvest** |
|  | **(56DAT)** | **(60DAT)** | **(64DAT)** | **(68DAT)** | **(72DAT)** | **(76DAT)** | **(80DAT)** | **(84DAT)** | **(89DAT)** |
| **D2** | 0±0 | 0.42±0.42 | 0±0 | 0.61±0.61 | 0±0 | 0±0 | 0±0 | 0.69±0.69 | 0±0 |
| **D3** | 0±0 | 0±0 | 0±0 | 0±0 | 0±0 | 0±0 | 0.69±0.69 | 0±0 | 0.36±0.36 |
| **M1** | 0±0 | 0±0 | 0±0 | 1.67±1.67 | 0±0 | 0±0 | 12.50±12.50 | 0±0 | 0±0 |
| **M4** | 0±0 | 0±0 | 0±0 | 8.30±4.83 | 5.64±2.30 | 9.69±4.68 | 4.47±3.14 | 6.20±4.17 | 3.59±1.96 |
| **M8** | 3.57±3.57 | 1.67±1.67 | 0±0 | 2.33±1.53 | 3.76±2.78 | 3.22±1.95 | 3.80±3.80 | 6.09±3.96 | 1.77±1.33 |
| **DLP** | 100.00±0 | 80.35±6.60 | 72.53±3.66 | 76.96±5.60 | 73.92±2.50 | 76.23±10.11 | 81.86±4.99 | 77.52±5.36 | 60.40±1.97 |
| **Mara** | 85.70±2.43 | 81.00±7.10 | 72.22±3.21 | 74.86±13.14 | 72.12±7.06 | 88.42±4.32 | 87.36±2.51 | 83.20±2.16 | 74.50±4.79 |
| **Mamburao** | 50.00±28.86 | 83.92±6.83 | 47.50±2.50 | 90.00±5.77 | 63.09±22.11 | 84.00±5.83 | 83.96±7.98 | 76.06±18.78 | 68.05±11.19 |

^1^Mean of four replicates, 9 harvest periods

Data were taken from nine (9) harvest periods starting when 50% of the plants in all entries were fruiting; available fruits were harvested only from the 16 plants in the two inner rows/plot

**S4B Table.** **Mean ± SEM of percentage EFSB fruit damage of Bt OP lines and non-Bt eggplants comparators.** Percentage EFSB fruit damage^1^ every harvest period (days after transplanting, DAT) in Bt eggplant OP lines containing event ‘EE-1’ and conventional non-Bt eggplant comparators .Trial 2. Bgy. Paitan, Sta. Maria, Pangasinan, Philippines

| **Entry** | **Percentage (%) EFSB fruit damage per plot** | | | | | | |
| --- | --- | --- | --- | --- | --- | --- | --- |
|  | **1st Harvest** | **2nd Harvest** | **3rd Harvest** | **4th Harvest** | **5th Harvest** | **6th Harvest** | **7th Harvest** |
|  | **(73DAT)** | **(78DAT)** | **(81DAT)** | **(85DAT)** | **(89DAT)** | **(93DAT)** | **(96DAT)** |
| **D2** | 0±0 | 0±0 | 0±0 | 0.32±0.32 | 0.86±0.86 | 0±0 | 5.69±1.06 |
| **D3** | 0±0 | 0±0 | 2.17±2.173 | 1.67±1.67 | 1.27±0.79 | 0.79±0.51 | 3.76±1.23 |
| **M1** | 0±0 | 0±0 | 0±0 | 0.37±0.37 | 1.07±0.38 | 1.03±0.60 | 1.42±1.42 |
| **M4** | 0±0 | 0±0 | 0±0 | 0±0 | 0±0 | 0±0 | 0±0 |
| **M8** | 0±0 | 0±0 | 0±0 | 0±0 | 1.34±0.54 | 2.01±1.17 | 5.51±1.51 |
| **DLP** | 100.00±0 | 93.75±6.25 | 91.66±8.33 | 97.22±2.78 | 96.77±1.86 | 96.91±1.78 | 90.49±4.24 |
| **Mara** | 90.00±10.00 | 71.42±24.04 | 78.46±12.49 | 80.13±7.16 | 86.67±3.74 | 93.08±2.64 | 95.83±4.17 |
| **Mamburao** | 100.00±0 | 100.00±0 | 100.00±0 | 93.88±3.76 | 97.60±1.43 | 97.46±1.50 | 92.30±7.70 |
| **Entry** | **8th Harvest** | **9th Harvest** | **10th Harvest** | **11th Harvest** | **12th Harvest** | **13th Harvest** |  |
|  | **(101DAT)** | **(106DAT)** | **(109DAT)** | **(113DAT)** | **(117DAT)** | **(121DAT)** |  |
| **D2** | 2.84±1.34 | 0±0 | 0±0 | 2.27±1.32 | 2.70±1.56 | 3.75±3.75 |  |
| **D3** | 1.28±1.28 | 2.44±1.41 | 2.44±1.41 | 2.61±1.52 | 2.61±1.53 | 1.61±1.61 |  |
| **M1** | 0.61±0.61 | 0.96±0.96 | 0.96±0.96 | 0±0 | 0±0 | 1.65±1.12 |  |
| **M4** | 1.47±1.07 | 0±0 | 0±0 | 1.07±0.71 | 0.72±0.42 | 0.37±0.37 |  |
| **M8** | 2.03±0.79 | 2.79±1.61 | 2.79±1.61 | 0±0 | 0.44±0.44 | 2.16±1.00 |  |
| **DLP** | 87.58±5.40 | 91.66±4.81 | 91.66±4.81 | 89.46±4.29 | 87.06±8.61 | 95.83±4.17 |  |
| **Mara** | 90.67±3.72 | 67.50±13.01 | 65.42±14.87 | 67.06±4.12 | 86.82±7.061 | 91.39±2.06 |  |
| **Mamburao** | 75.00±25.00 | 91.66±5.89 | 94.34±3.48 | 89.88±6.02 | 70.83±23.93 | 90.62±8.05 |  |

^1^Mean of four replicates; 13 harvest periods

Data were taken from 13 harvest periods starting when 50% of the plants in all entries were fruiting; available fruits were harvested only from the 16 plants in the two inner rows/plot

**S4C Table**. **Mean ± SEM of percentage EFSB fruit damage of Bt OP lines and non-Bt eggplants comparators.** Percentage EFSB fruit damage^1^ every harvest period (days after transplanting, DAT) in Bt eggplant OP lines containing event ‘EE-1’ and conventional non-Bt eggplant comparators .Trial 3. Bgy. Paitan, Sta. Maria, Pangasinan, Philippines

| **Percentage (%) EFSB fruit damage per plot** | | | | | | | | | |
| --- | --- | --- | --- | --- | --- | --- | --- | --- | --- |
| **Entry** | **1st Harvest** | **2nd Harvest** | **3rd Harvest** | **4th Harvest** | **5th Harvest** | **6th Harvest** | **7th Harvest** | **8th Harvest** | **9th Harvest** |
|  | **(80DAT)** | **(84DAT)** | **(87DAT)** | **(91DAT)** | **(94DAT)** | **(98DAT)** | **(101DAT)** | **(105DAT)** | **(108DAT)** |
| **D2** | 0±0 | 0.58±0.58 | 0±0 | 0±0 | 0±0 | 0±0 | 0±0 | 0±0 | 15.00±15.00 |
| **M1** | 0±0 | 0±0 | 0±0 | 0±0 | 0±0 | 0±0 | 0±0 | 0±0 | 0±0 |
| **M8** | 0±0 | 0±0 | 2.50±2.50 | 0±0 | 0±0 | 2.63±2.63 | 0±0 | 0±0 | 0±0 |
| **DLP** | 33.33±20.41 | 90.91±9.09 | 46.43±17.61 | 78.75±8.26 | 63.75±21.92 | 78.98±10.95 | 72.91±14.18 | 51.60±11.45 | 22.81±10.33 |
| **Mara S1** | 61.19±9.87 | 73.12±13.04 | 66.66±23.57 | 35.00±23.62 | 58.33±22.04 | 48.21±7.36 | 49.85±16.81 | 23.52±11.21 | 25.12±7.13 |
| **Mara S2** | 25.89±17.70 | 77.44±3.61 | 69.36±10.29 | 76.74±13.92 | 55.56±11.53 | 65.44±5.74 | 31.94±12.65 | 33.31±11.83 | 13.33±5.65 |
| **Mamburao** | 95.83±4.17 | 94.44±5.56 | 53.03±18.11 | 70.83±19.69 | 67.91±6.574 | 69.96±5.49 | 44.44±16.19 | 37.17±15.32 | 27.38±16.53 |

^1^Mean of four replicates, 9 harvest periods

Data were taken from 9 harvest periods starting when 50% of the plants in all entries were fruiting; available fruits were harvested only from the 16 plants in the two inner rows/plot
