# Supplementary material for: Field Performance of Bt Eggplants (Solanum melongena L.) in the Philippines: Cry1Ac Expression and Control of the Eggplant Fruit and Shoot Borer (Leucinodes orbonalis Guenée)
Source: PLoS One. 2016 Jun 20;11(6):e0157498. doi: 10.1371/journal.pone.0157498 (PMC4913932; doi:10.1371/journal.pone.0157498)
Supplement: S5 Table — A: Trial 1; B: Trial 2; C: Trial 3. CY 2010–12, Sta. Maria, Pangasinan, Philippines. (DOCX) [file pone.0157498.s005.docx]

**S5A Table.** **S5 Table. Mean ± SEM EFSB larval counts in fruits of Bt OP lines and non-Bt eggplants comparators.** EFSB larval counts^1^ in fruits every harvest period (days after transplanting, DAT) in Bt eggplant OP lines containing event ‘EE-1’ and conventional non-Bt eggplant comparators. Trial 1. Bgy. Paitan, Sta. Maria, Pangasinan. Philippines

| **Entry** | **EFSB larval counts (no. per plot)** | | | | | | | | |
| --- | --- | --- | --- | --- | --- | --- | --- | --- | --- |
|  | **1st Harvest** | **2nd Harvest** | **3rd Harvest** | **4th Harvest** | **5th Harvest** | **6th Harvest** | **7th Harvest** | **8th Harvest** | **9th Harvest** |
|  | **(56DAT)** | **(60DAT)** | **(64DAT)** | **(68DAT)** | **(72DAT)** | **(76DAT)** | **(80DAT)** | **(84DAT)** | **(89DAT)** |
| **D2** | 0±0 | 0.25±0.13 | 0±0 | 0.25±0.13 | 0±0 | 0±0 | 0±0 | 0.25±0.13 | 0±0 |
| **D3** | 0±0 | 0±0 | 0±0 | 0±0 | 0±0 | 0±0 | 0.25±0.13 | 0±0 | 0.25±0.13 |
| **M1** | 0.25±0.13 | 0.25±0.13 | 0±0 | 0.50±0.25 | 0±0 | 1.25±0.63 | 2.25±1.13 | 0.50±0.25 | 0.50±0.25 |
| **M4** | 0±0 | 0±0 | 0±0 | 0±0 | 0±0 | 0±0 | 0.25±0.13 | 0±0 | 0±0 |
| **M8** | 0±0 | 0±0 | 0±0 | 1.00±0.50 | 0.75±0.38 | 1.00±0.50 | 0.75±0.38 | 0.75±0.38 | 1.00±0.50 |
| **DLP** | 5.25±2.63 | 4.50±2.25 | 2.75±1.38 | 6.00±3.00 | 7.25±3.63 | 13.75±6.88 | 23.25±11.63 | 5.75±2.88 | 8.25±4.13 |
| **Mara** | 8.00±4.00 | 6.00±3.00 | 5.75±2.88 | 4.25±2.13 | 11.00±5.50 | 20.25±10.13 | 16.70±8.38 | 5.50±2.75 | 5.50±2.75 |
| **Mamburao** | 2.25±1.13 | 3.00±1.50 | 1.00±0.50 | 1.75±0.88 | 2.75±1.38 | 12.25±6.13 | 12.50±6.25 | 8.75±4.38 | 3.00±1.50 |

^1^Mean of four replicates, 9 harvest periods

Data were taken from nine (9) harvest periods starting when 50% of the plants in all entries were fruiting; available fruits were harvested only from the 16 plants in the two inner rows/plot

**S5B Table.** **S5 Table. Mean ± SEM EFSB larval counts in fruits of Bt OP lines and non-Bt eggplants comparators.** EFSB larval counts^1^ in fruits every harvest period (days after transplanting, DAT) in Bt eggplant OP lines containing event ‘EE-1’ and conventional non-Bt eggplant comparators. Trial 2. Bgy. Paitan, Sta. Maria, Pangasinan. Philippines

| **Entry** | **EFSB larval counts (no. per plot)** | | | | | | |
| --- | --- | --- | --- | --- | --- | --- | --- |
|  | **1st Harvest** | **2nd Harvest** | **3rd Harvest** | **4th Harvest** | **5th Harvest** | **6th Harvest** | **7th Harvest** |
|  | **(73DAT)** | **(78DAT)** | **(81DAT)** | **(85DAT)** | **(89DAT)** | **(93DAT)** | **(96DAT)** |
| **D2** | 0±0 | 0±0 | 0±0 | 0.25±0.25 | 0.25±0.25 | 0±0 | 1.00±0.41 |
| **D3** | 0±0 | 0±0 | 0.50±0.50 | 0.50±0.50 | 0.75±0.48 | 0.25±0.25 | 0.75±0.48 |
| **M1** | 0±0 | 0±0 | 0±0 | 0±0 | 0.75±0.25 | 0.25±0.25 | 0±0 |
| **M4** | 0±0 | 0±0 | 0±0 | 0±0 | 0±0 | 0±0 | 0±0 |
| **M8** | 0±0 | 0±0 | 0±0 | 0±0 | 1.00±0.41 | 1.75±1.05 | 1.25±0.25 |
| **DLP** | 5.00±1.58 | 3.25±0.85 | 2.25±0.63 | 9.25±2.21 | 40.50±10.56 | 36.25±5.94 | 9.75±3.12 |
| **Mara** | 2.25±0.48 | 2.25±1.11 | 3.50±1.50 | 18.00±3.19 | 27.50±4.57 | 17.25±4.21 | 10.25±3.38 |
| **Mamburao** | 2.00±0.41 | 4.75±0.75 | 3.25±1.18 | 21.75±7.15 | 22.75±0.49 | 23.75±0.86 | 10.75±3.75 |
| **Entry** | **8th Harvest** | **9th Harvest** | **10th Harvest** | **11th Harvest** | **12th Harvest** | **13th Harvest** |  |
|  | **(101DAT)** | **(106DAT)** | **(109DAT)** | **(113DAT)** | **(117DAT)** | **(121DAT)** |  |
| **D2** | 1.00±0.41 | 2.25±1.32 | 0±0 | 1.00±0.58 | 0.50±0.29 | 0.50±0.50 |  |
| **D3** | 1.25±1.25 | 1.00±1.00 | 0.25±0.25 | 1.00±0.58 | 0.50±0.29 | 0.50±0.50 |  |
| **M1** | 0±0 | 0.25±0.25 | 0±0 | 0±0 | 0±0 | 0.25±0.25 |  |
| **M4** | 0.25±0.25 | 0±0 | 0±0 | 0.75±0.48 | 0±0 | 0.25±0.25 |  |
| **M8** | 0.75±0.48 | 0.50±0.50 | 0.50±0.29 | 0±0 | 0.25±0.25 | 0.50±0.29 |  |
| **DLP** | 14.00±4.14 | 10.50±3.12 | 23.00±6.26 | 21.25±9.31 | 15.00±3.69 | 20.00±7.52 |  |
| **Mara** | 15.75±4.61 | 7.75±3.75 | 16.25±6.90 | 12.50±2.87 | 21.75±6.05 | 22.75±4.37 |  |
| **Mamburao** | 13.75±6.16 | 7.50±2.60 | 14.50±7.29 | 18.25±3.68 | 11.00±6.56 | 18.00±5.82 |  |

^1^ Mean of 4 replicates per harvest, 13 harvests

Data were taken from 13 harvest periods starting when 50% of the plants in all entries were fruiting; available fruits were harvested only from the 16 plants in the two inner rows/plot

**S5C Table.** **S5 Table. Mean ± SEM EFSB larval counts in fruits of Bt OP lines and non-Bt eggplants comparators.** EFSB larval counts^1^ in fruits every harvest period (days after transplanting, DAT) in Bt eggplant OP lines containing event ‘EE-1’ and conventional non-Bt eggplant comparators. Trial 3. Bgy. Paitan, Sta. Maria, Pangasinan. Philippines

| **Entry** | **EFSB larval counts (no. per plot)** | | | | | | | | |
| --- | --- | --- | --- | --- | --- | --- | --- | --- | --- |
|  | **1st Harvest** | **2nd Harvest** | **3rd Harvest** | **4th Harvest** | **5th Harvest** | **6th Harvest** | **7th Harvest** | **8th Harvest** | **9th Harvest** |
|  | **(80DAT)** | **(84DAT)** | **(87DAT)** | **(91DAT)** | **(94DAT)** | **(98DAT)** | **(101DAT)** | **(105DAT)** | **(108DAT)** |
| **D2** | 0±0 | 0.25±0.25 | 0±0 | 0±0 | 0±0 | 0±0 | 0±0 | 0±0 | 0.25±0.25 |
| **M1** | 0±0 | 0±0 | 0±0 | 0±0 | 0±0 | 0±0 | 0±0 | 0±0 | 0±0 |
| **M8** | 0±0 | 0±0 | 0±0 | 0±0 | 0±0 | 0.50±0.50 | 0±0 | 0±0 | 0±0 |
| **DLP** | 1.25±1.25 | 2.75±0.75 | 4.00±1.47 | 3.00±1.73 | 1.75±0.85 | 4.00±2.12 | 2.25±1.25 | 2.50±0.96 | 1.25±0.75 |
| **Mara S1** | 3.00±0.71 | 2.75±0.48 | 2.75±1.38 | 0.50±0.50 | 2.50±0.87 | 2.75±0.85 | 0.75±0.480 | 1.50±0.65 | 0.75±0.48 |
| **Mara S2** | 1.50±0.96 | 10.75±0.95 | 5.50±1.56 | 3.00±0.91 | 4.00±0.41 | 9.75±1.80 | 1.75±1.03 | 1.50±0.96 | 1.75±1.11 |
| **Mamburao** | 3.00±1.41 | 4.00±2.71 | 3.50±2.65 | 5.75±2.75 | 2.50±1.91 | 6.00±0.71 | 1.50±1.29 | 1.50±1.73 | 0.50±1.00 |

^1^ Mean of 4 replicates per harvest, 9 harvests

Data were taken from nine (9) harvest periods starting when 50% of the plants in all entries were fruiting; available fruits were harvested only from the 16 plants in the two inner rows/plot
